# Biological network topology features predict gene dependencies in cancer cell-lines

**DOI:** 10.1093/bioadv/vbac084

**Published:** 2022-11-10

**Authors:** Graeme Benstead-Hume, Sarah K Wooller, Joanna Renaut, Samantha Dias, Lisa Woodbine, Antony M Carr, Frances M G Pearl

**Affiliations:** Bioinformatics Lab, School of Life Sciences, University of Sussex, Brighton BN1 9QJ, UK; Division of Cancer Biology, The Institute of Cancer Research, London SW3 6JB, UK; Bioinformatics Lab, School of Life Sciences, University of Sussex, Brighton BN1 9QJ, UK; Bioinformatics Lab, School of Life Sciences, University of Sussex, Brighton BN1 9QJ, UK; Genome Damage and Stability Centre, University of Sussex, Brighton BN1 9RQ, UK; Genome Damage and Stability Centre, University of Sussex, Brighton BN1 9RQ, UK; Genome Damage and Stability Centre, University of Sussex, Brighton BN1 9RQ, UK; Bioinformatics Lab, School of Life Sciences, University of Sussex, Brighton BN1 9QJ, UK

## Abstract

**Motivation:**

Protein–protein interaction (PPI) networks have been shown to successfully predict essential proteins. However, such networks are derived generically from experiments on many thousands of different cells. Consequently, conventional PPI networks cannot capture the variation of genetic dependencies that exists across different cell types, let alone those that emerge as a result of the massive cell restructuring that occurs during carcinogenesis. Predicting cell-specific dependencies is of considerable therapeutic benefit, facilitating the use of drugs to inhibit those proteins on which the cancer cells have become specifically dependent. In order to go beyond the limitations of the generic PPI, we have attempted to personalise PPI networks to reflect cell-specific patterns of gene expression and mutation. By using 12 topological features of the resulting PPIs, together with matched gene dependency data from DepMap, we trained random-forest classifiers (DependANT) to predict novel gene dependencies.

**Results:**

We found that DependANT improves the power of the baseline generic PPI models in predicting common gene dependencies, by up to 10.8% and is more sensitive than the baseline generic model when predicting genes on which only a small number of cell types are dependent.

**Availability and implementation:**

Software available at https://bitbucket.org/bioinformatics_lab_sussex/dependant2

**Supplementary information:**

Supplementary data are available at *Bioinformatics Advances* online.

## 1 Introduction

An essential gene is one on which a cell depends for cellular survival. However, the set of essential genes is context specific, depending on the cell type, genetic and epigenetic aberrations, and the environment the cell finds itself in. Different definitions and measurements of essentiality often have considerable overlap, but there are also large areas of disagreement (Bartha *et al.*, 2018; Eisenberg and Levanon, 2013).

During the process of carcinogenesis, the cell becomes addicted to oncogenes and tumour suppressor genes become inactivated. Consequently, the cell develops new gene dependencies as different genes become transiently essential to that cell as it evolves (Acencio *et al.*, 2009; Weinstein, 2002). These gene dependencies can provide opportunities for targeted treatments, as inhibiting proteins which are only essential in cancer cells can provide a therapeutic opportunity (Workman *et al.*, 2013).

There have been significant experimental efforts to identify and catalogue gene dependencies in cell-lines. Amongst these are a number of loss-of-function (LOF) screens (Ngo *et al.*, 2006) performed using both RNAi and CRISPR-Cas9 (Aguirre *et al.*, 2016; Aksoy *et al.*, 2014; Cheung *et al.*, 2011; Luo *et al.*, 2008; Marcotte *et al.*, 2012, 2016), which investigate the changes in cellular phenotype caused by systematically knocking genes out one by one, through down-regulation or disruption. Knock-downs or knock-outs that result in significantly deleterious phenotypes indicate that the respective gene may be essential in that cell-line.

A number of studies have reported off-target effects in loss of function screens, where genes other than the target are disrupted by certain RNAi or CRISPR treatments (Aguirre *et al.*, 2016; Birmingham *et al.*, 2006; Buehler *et al.*, 2012; Jackson and Linsley, 2004; Munoz *et al.*, 2016). In response to these challenges, Tsherniak developed DEMETER, an analytical framework to segregate the on- and off-target effects found in RNAi treatments (Tsherniak *et al.*, 2017). In addition, Meyers *et al.* developed the computational method, CERES, to estimate gene-dependency levels from CRISPR–Cas9 essentiality screens while accounting for copy number–specific effects. A gene dependency score was assigned to each gene in each cell-line, reflecting the probability that the cell-line depends on the gene (Meyers *et al.*, 2017).

Identifying core essential genes or disease-specific gene dependencies provides a better understanding of potential disease-specific targets. However, LOF screens are not readily available for the majority of individual cancer patients. Computational tools that predict cell-line specific gene dependencies from more readily available data such as mutations and gene expression, may offer new opportunities for tailored therapies (Benstead-Hume *et al.*, 2017; Charlton and Spicer, 2016).

There have been a number of successful attempts to predict common essential genes using biological network data in different contexts and in different organisms [for a review see Zhang* et al.*, (2016)]. These studies have used a range of different network data including protein–protein interaction (PPI) networks, transcriptional regulatory networks, gene co-expression networks, metabolic networks and networks that integrate two or more of the above. Due to data availability these studies have generally focused on model organisms including *Saccharomyces cerevisiae* (Acencio *et al.*, 2009; Chen and Xu, 2005; Saha and Heber, 2006), *Escherichia coli* (da Silva *et al.*, 2008; Hwang *et al.*, 2009) and on several other species of bacteria (Cheng *et al.*, 2014; Lu *et al.*, 2014; Plaimas *et al.*, 2010).

For the most part these studies employ similar methods; topology data is extracted from the biological networks and used as a feature-set to train machine learning models to identify essential genes. For example, Saha and Heber (2006) reported a Receiver Operating Characteristic (ROC) Area Under the Curve (AUC) of 82% using PPI network degree count and conservation score features, to classify ∼2200 essential genes in *S.cerevisiae*, and da Silva *et al.* (2008) reported accuracy, using F-measure scores, of 83.4% for essential gene predictions and 79.7% for non-essential gene prediction in *E.coli.* Comparable predictions for *Homo sapiens* have recently been reported (Dai *et al.*, 2020; Schapke *et al.*, 2021; Zhang *et al.*, 2020).

Previous computational studies have generally assumed that genes are either essential or not, and have used a static version of the known PPI network. However, observations made by Roumeliotis *et al.* (2017) suggest the effect of genetic variations can be transmitted from directly affected proteins to distant gene products through protein interaction pathways, which suggests that a more dynamic model of the PPI may be required to capture gene dependencies in highly mutated cancer cells.

In cancer cells, mutations and changes in expression level and/or copy number of one gene, can impact the function of proteins encoded by other genes, thereby altering the underlying connectivity of the PPI network. Topological network features such as eigen-centrality and betweenness are effective at capturing long-distance changes in networks, which suggests that the inclusion of genetic alterations to the traditional PPI network model could be an appropriate way of predicting altered and novel dependencies.

In this study, we use recent cell-line specific gene dependency data along with data from PPI networks, to build models able to identify novel cell-line specific gene dependencies. To do this we model genetic alterations in specific cell-lines by perturbing their respective PPI networks. We explore the viability of identifying cell-line specific gene dependencies both within and between various human cancer cell-lines using this perturbed PPI network data. Finally, we introduce DependANT, a classifier trained to predict cell-line specific gene dependencies using both generic and perturbed PPI network data, with the aim of providing a low-cost approach to identifying personalised cancer drug targets without the need for experimental dependency screening.

## 2 Methods

We generated a baseline human PPI network, a network of known physical protein interactions in which each node (7262 nodes) represents a protein and each edge (∼60 000 edges) a known physical protein interaction. The PPI data was sourced from the STRING database (v.10) (von Mering *et al.*, 2005) and filtered to include only human interactions with an experimental score higher than 80, to ensure reliability. The Ensembl protein IDs (ENSPs) in this dataset were converted to their respective gene IDs (ENSGs) using Ensembl data (Hubbard *et al.*, 2002). R (version 3.4.0) and the igraph package (version 1.1.2) (Csárdi and Nepusz, 2006) was used to produce a network model of the PPI data for each cell-line and calculate each topological feature.

To establish a baseline performance for classification we sourced gene dependency data published in Meyers *et al.* via DepMap Public 18Q3 (Tsherniak *et al.*, 2017). These data provide a matrix of probabilities that a cell line is dependent on a gene, independent of copy number. We selected 39 cell lines, 19 breast, 11 kidney and 9 pancreas based on data availability. In the analysis following we use the term ‘cell-essential gene’ to refer to a gene which, in the context of a particular cell-line has a probability >0.65 that the cell is dependent on the gene. Genes were identified as pan-essential if they were predicted to be a cell-essential gene in all the 39 cell lines analysed.

Twelve topological features for each node of the PPI were calculated: betweenness; constraint; closeness; coreness; degree; eccentricity; eigen centrality; hub score; neighbourhood *n* size (for *n* = 1,2,6); page rank (Table 1). After normalization, where each feature score was independently scaled between 0 and 1, these were used as features in our machine learning models. For each of the 39 cell-lines we optimized and trained a random forest classifier using a balanced set of nodes labelled as dependent and non-dependent, averaging around 1000 dependent/non-cell-essential genes for each cell line, and holding out 20% of the data as a test set, with a further 20% for validation.

**Table 1.** List of graph topology features data with descriptions

Feature name	Description
Betweenness	The number of shortest paths in the entire graph that pass through the node.
Constraint	A measure of how many of a node’s connections are focused on single cluster of neighbours.
Closeness	The number of steps required to reach all other nodes from a given node.
Coreness	Whether a node is part of the k-core of the full graph, the k-core being a maximal sub-graph in which each node has at least degree k.
Degree	The number of edges coming in to or out of the node.
Eccentricity	The shortest path distance from the node farthest from the given node.
Eigen centrality	A measure of how well-connected a given node is to other well-connected nodes.
Hub score	Related to the concepts of hubs and authorities, the hub score is a measure of how many well linked hubs the nodes is linked to.
PageRank (PRPACK)	Another measure of how well-connected a given node is to other well-connected nodes.
Neighbourhood *n* size (1, 2 and 6 steps)	The number of nodes within n steps of a given node for n of 1, 2 and 6.

*Note:* Features were extracted using R igraph package (version 1.1.2) from protein interaction network data.

We validated the model on each of our cell-lines, using both training data and validation data extracted from the same single cell-line. Each trial was repeated 10 times using the base PPI model. To understand how well the classifiers perform and generalize to different cell types, we tested each of the classifiers on unseen, balanced data from three different sets: the same cell-line; different cell-lines of the same tissue type; and from cell-lines originating from different tissue types.

### 2.1 Perturbing the PPI

The base PPI network was then weighted to more accurately represent the differences in the 39 cell-lines. Mutations such as frameshift, insertions and deletions (indels) or nonsense substitutions were labelled as LOF. For missense mutations the pathogenic mutations were identified using the FATHMM algorithm (Shihab *et al.*, 2013), and classified as gain-of-function (GOF) or LOF depending on whether they came from oncogenes or tumour suppressor genes. Nodes that represented genes with inactivating mutations were removed from the PPI network.

As well as removing inactivated nodes we also weighted edges. We assume that genes with either GOF mutations and/or high expression levels have greater impact than in the baseline network. To model this, we give the edges from the corresponding proteins in the PPI network low weights. This increases the flow of information and so strengthens the impact of the gene. Edges from nodes with low expression levels have high weights, reducing the flow of information and thus weakening the impact. To do this, mutation and gene expression data from Meyer *et al.* via project Achilles (Tsherniak *et al.*, 2017) was used to establish the weights, as follows. For each gene pair we replaced the unweighted bidirectional edge with two weighted unidirectional edges, so that as gene expression (*g*) tends to 0 the weight (*w*) tends to 1, and as g tends to infinity, w tends to 0. For genes subject to a gain of function mutation we multiplied the gene expression by 10 before calculating the weight (*w*) as a function of the gene expression g. To provide the properties described above we used the function

w=0.5-0.5 tanh⁡(log⁡(g+1e-10)),

where *g* is the expression in transcripts per million. To focus on the differences in gene dependency between tissue types we removed genes that are either almost always essential or almost never essential (variation in gene dependency across all cell lines <0.1). Features for the classification algorithm were recalculated for each individual cell-line weighted by the edge scores for each node. To improve performance in cross cell-line classification each cell-line’s feature set was normalized, i.e. each feature was independently scaled between 0 and 1 (Jacunski *et al.*, 2015). To ensure unbiased validation we withheld 20% of this data to be used as a test set.

To measure performance across cell-lines originating from the same tissue type and the predictive performance between tissue types, we used the training sets that were already generated for each cell-line to train our classifiers, and systematically validated them against each other cell-lines test set. To ensure that our models were not being biased by genes that were present in both training and test sets we ensured that any genes present in the training set were removed from the active test set.

We predicted both the probability that unseen proteins from the same cell-line were cell-essential and that unseen proteins from other cell lines were cell-essential. To predict cell-essential genes in unlabelled cell-lines we first concatenated all training data into one large, labelled training dataset. We produced a number of feature sets for cell-lines that were not included in the original training data and predicted cell-essential genes in these unlabelled cell-lines based on a model trained on the pan-cancer set.

R script used for analysis is available: https://bitbucket.org/bioinformatics_lab_sussex/dependant2

### 2.2 Experimental validation

We validated our predictions experimentally using the unlabelled breast cancer cell-line, MCF7 as it is not featured in our training data, readily available and has a good class balance for predictions on genes featured as part of the available DDR gene library. We performed a high-throughput siRNA screen. MCF7 cells (validated by ATCC STR.V profiling) were grown in minimal essential medium (MEM) supplemented with 10% foetal calf serum, penicillin/streptomycin and L-glutamine at 37°C and 5% CO_2_. Cells were reverse transfected with library siRNA using lipofectamine RNAiMAX (as per the manufacturer’s instructions) in black 96 well plates. Plates were incubated at 37°C, 5% CO_2_ for 72 h. CellTitre-Blue was added to determine cell viability, plates were analysed using a plate reader at 560/590 nm.

### 2.3 Druggability analysis

Druggability annotation was performed using CanSAR Black’s cancer protein annotation tools (Coker *et al.*, 2019). We designated any genes with a ‘nearest drug target’ score of 100% as a known drug target. We also identified a set of ‘druggable genes’. These were genes whose protein products were predicted to be drug targets using a structural druggability algorithm on the human protein structure (Burley *et al.*, 2022).

## 3 Results

### 3.1 Predicting gene dependencies using PPI network topological features

In total, we used 39 cell-lines with dependency data, mutational data and gene expression data. Across these cell-lines 4030 genes had dependency scores >0.65 in one or more cell-line. We refer to these as cell-essential genes.

### 3.2 Base PPI network parameter data predicts pan-cell-line essential genes

We first predicted genes which are cell-essential in all the cell-lines, i.e. pan-cell-line essential genes. To create a baseline score, we performed the prediction on a base PPI model with randomized edges. With a balanced class distribution of positive and negative labelled DepMap genes, we achieved an AUC ROC score of 0.520 (SD 0.082, *n* = 10). Significance scores below are based on this baseline distribution.

On average the baseline PPI networks predicted pan-cell-line essential genes with an AUC ROC score of 0.765 (SD 0.024, *P* < 0.0001) when the classifiers were tested on genes from the same cell-line. This dropped slightly to an average of AUC ROC 0.758 (SD 0.007, *P* < 0.0001) when they were tested on genes from other cell-lines. Differences between the tissue types are shown in Table 2.

**Table 2.** Mean model performance when using dependencies from one cell line to predict those in another cell line of the same and different tissue types

	Test data (ROC)		
Training data	Breast	Kidney	Pancreas
Breast	0.761 (SD 0.005)	0.759 (SD 0.006)	0.761 (SD 0.01)
Kidney	0.758 (SD 0.007)	0.755 (SD 0.008)	0.758 (SD 0.01)
Pancreas	0.760 (SD 0.011)	0.752 (SD 0.01)	0.754 (SD 0.012)

### 3.3 Modelling cell-lines with biological network and genetic alteration data

**Fig. 1.**
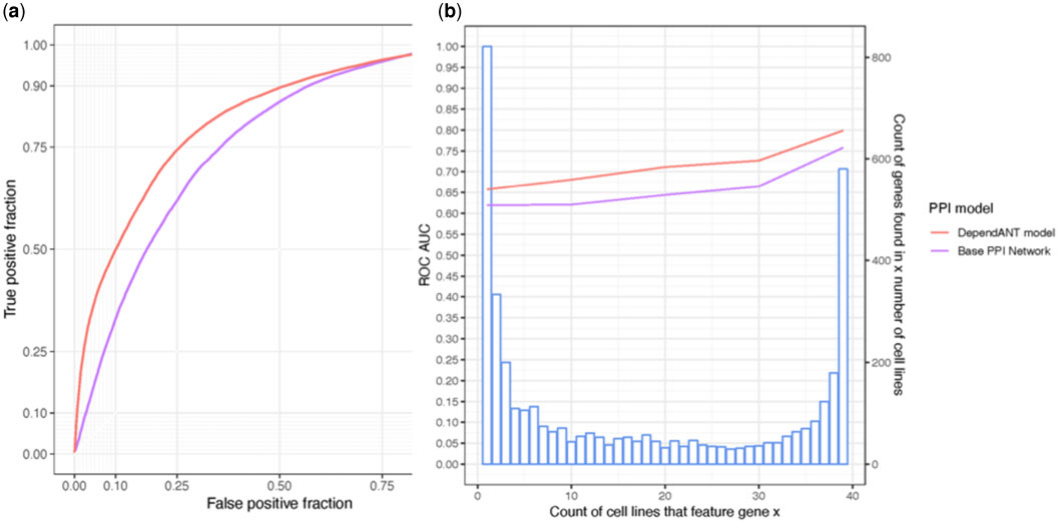
(**a**) AUC ROC plot for the base PPI model is shown in purple (lower line). The red line shows the improvement in prediction scores when the modified DependANT PPI model is used. (**b**) A comparison of how well the DependANT and base PPI models predict common and rare cell-essential genes. The blue bars represent the number of cell lines in which a gene is essential in. For example, 200 genes are reported to be cell-essential genes in exactly three cell lines

The PPI networks were modified using the mutation and gene expression data to create the DependANT classifier (see Supplementary Table S2). Again using balanced class validation data; DependANT improved the average predictive power of the baseline model by 5.7% (from a mean AUC ROC score of 0.758 to 0.801). Predictive power was highest when classifying pan-cell-line essential genes. On average the classifiers using modified PPI data and raw gene expression data scored a mean AUC ROC of 0.812 (SD 0.023, *P* < 0.0001) when predicting pan-cell-line essential genes compared to the base model’s performance of AUC ROC 0.765 (see Fig. 1a).

However, the therapeutic benefit of predicting gene dependencies is in predicting novel dependencies that only arise in a few cell-lines. To investigated how well our classifiers do under these circumstances, we trained DependANT on all cell-lines and then validated the models on test sets filtered for the rarity of the novel gene dependencies being predicted (see Fig. 1b). 580 of the total 4030 (∼14.3%) cell-essential genes in our training data had high scores in all 39 cell-lines, whereas 1606 (∼39.9%) were cell-essential genes in 20 or less of the 39 cell-lines. As expected, performance of both the baseline PPI models and DependANT fell when predicting genes that are only rarely dependencies. However, in each case DependANT significantly outperforms the baseline models (see Table 3) by broadly similar amounts, and thus proportionately larger amounts for the rarer dependencies. Model performance is also shown. The purple line shows the ROC AUC for the baseline PPI for different levels of rarity of cell-essential genes. The predictions being most reliable for genes that were essential in all the cell-lines studied (39). The red line shows the general improved performance of DepentANT which uses weighted PPIs.

**Table 3.** Model performance across gene dependency rarity intervals shows the general improved performance of the modified PPI against the baseline

	DependANT	Baseline PPI	% difference
All cell-lines	0.801	0.758	5.7
<30 cell-lines	0.727	0.665	9.3
<20 cell-lines	0.711	0.644	10.4
<10 cell-lines	0.681	0.621	9.7
1 cell-line	0.66	0.615	7.3

### 3.4 Feature importance

Twelve topological features were included within the dependency classifiers. To understand whether or not these features provide predictive power we plotted the distribution of features’ values for genes with a dependency score above the 0.65 cut-off against those genes with a lower dependency score.

**Fig. 2.**
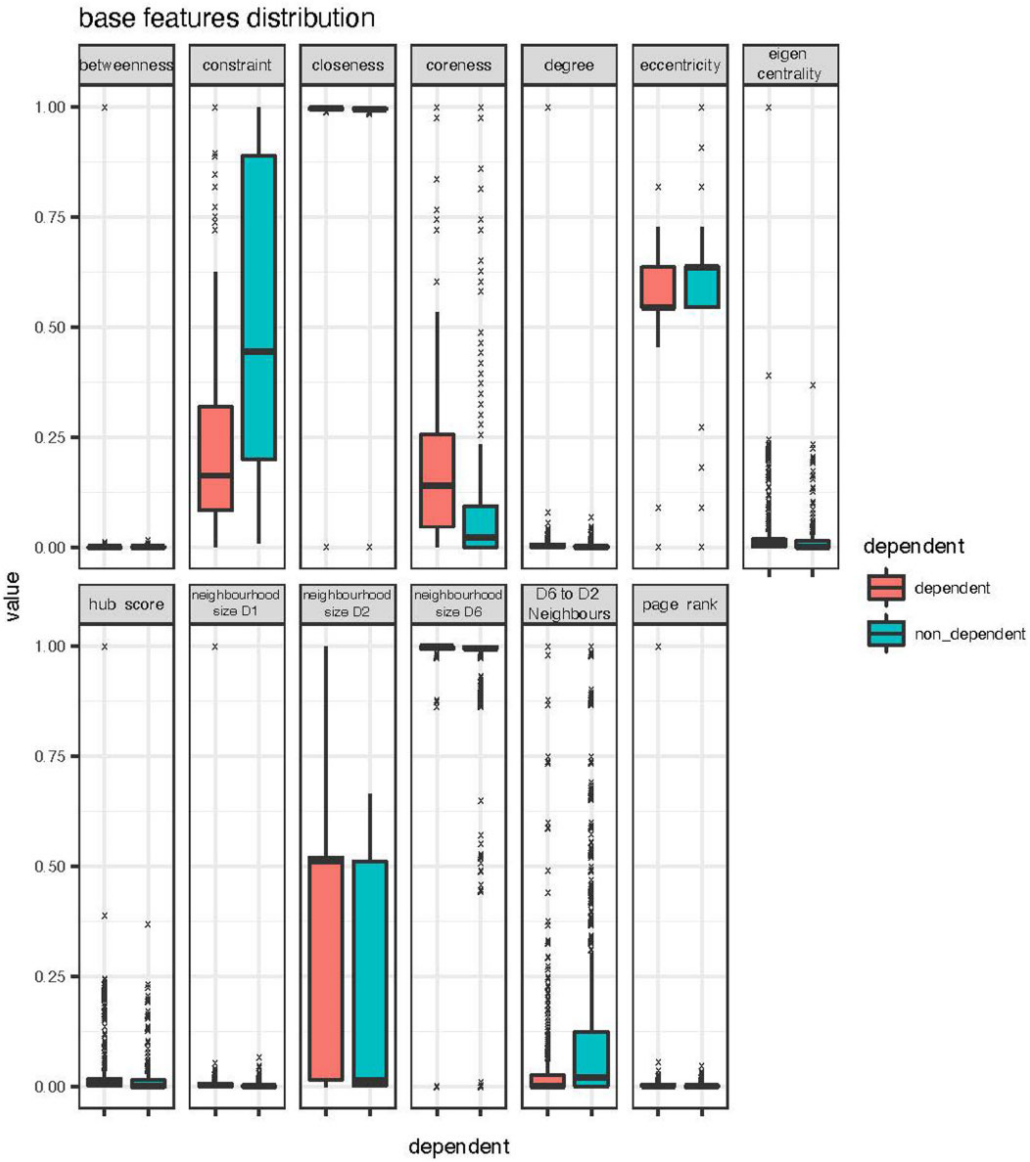
Feature distributions between dependent (red, left-hand side) and non-dependent (green, right-hand side) gene classes show differences between the classes for the betweenness, constraint, eigen centrality and hub score features

As Figure 2 shows, the two distributions are distinctive for features measuring betweenness, constraint, eigen centrality and hub-score features. This suggests that these features should provide predictive power.

To quantify the predictive power of each feature we performed a leave-one-out analysis, iteratively omitting each feature in turn and measuring the resulting mean decrease in accuracy, across all tree permutations in a random forest. We found that features (such as page rank and eigen centrality) that measure the connectivity of a gene, perform better than degree centrality. Eccentricity, which measures how close a node is to the centre of the network, performs badly across all models.

As expected, the difference in mean value for each feature across cell-essential and non-cell-essential genes is fairly well mirrored in these importance scores: features with values that vary more between cell-essential and non-cell-essential genes provided more predictive power. Pagerank and constraint showed a noticeable differentiation between classes, while the differentiation between classes for eigen centrality and hubscore features were not as prominent (see Supplementary Fig. S1).

### 3.5 Our models are fairly robust to PPI networks incompleteness

Current PPI network models are both incomplete and suffer from ascertainment bias in that some proteins are better studied than others (Huttlin *et al.*, 2017; Mosca *et al.*, 2013; Rolland *et al.*, 2014). In order to quantify how the incomplete nature of the PPI networks affects the robustness of our models, we repeated our classification pipelines with revised PPI networks in which a randomly selected 25% of the data forming the original network was withheld. We observed minimal loss of predictive power in the network derived from the randomly withheld dataset AUC ROC 0.78 (SD 0.011) compared to our raw expression cross cell-line model AUC ROC 0.801 (SD 0.006).

We conclude that while increasing completeness of a PPI network may improve its predictive performance, our current models are fairly resilient to the incomplete nature of the currently available PPI network data.

### 3.6 Creating a pan tissue cell-line training set

To maximize the amount of training data available for use by our classifiers for the prediction of gene dependencies in previously unlabelled cell-lines, we concatenated all available features/labels from all tissue types into one super set. In an attempt to estimate how well this concatenated data performs for the prediction of gene dependency in unlabelled datasets, we once more validated each of our individual test sets based on models trained using our super training set.

We found that our super training set classified gene dependencies across all cell-lines with an AUC ROC of 0.843 (SD 0.012), a further improvement on the mean individual cell AUC ROC score of 0.801 (SD 0.006). This model provided the most predictive power and as such represents the most suitable available for predicting gene dependencies in cell-lines with no prior labelling as discussed below.

### 3.7 Experimentally validating gene dependencies in previously unlabelled cancer cell-lines

The MCF7 breast cell line was chosen to experimentally validate the DependANT classifier due to the availability of its mutational and expression data. We used our pan-tissue training set to train our classifiers and produced a full set of predictions for the MCF7 breast cell-line.

Survival screens focusing on a library of 240 genes involved in the DNA damage response (DDR) were repeated in triplicate for the MCF7 breast cell-line. Cell viability for knockdown of each of the genes was reported using a z-score: a positive number indicates that viability increased with the knockdown of the gene, whilst a negative z-score indicates a decrease in viability.

The variance of results across all three repeats was high, which may have been due to the choice of library (see Supplementary Table S2). The loss of genes involved in the DDR can often lead to genomic instability in a cell, with consequential mutations in other genes or loci. Knocking out a single gene (e.g. MSH3) can thus cause changes in the function of additional genes, resulting in a complex pattern of dependencies. We then compared the z-scores with the cell-line essentiality predictions (see Supplementary Figs S2–S4). We classify a gene as ‘predicted cell-essential’ if the likelihood is >0.85 and ‘predicted non-cell-essential’ if the likelihood is <0.15. This gives an accuracy of 0.64 with a sensitivity of 0.73 and a false discovery rate of 0.38 based on experimental validation for the MCF7 cell-line. Of the 10 genes with the highest cell-essentiality score (ce-score), eight also have a mean negative z-score (see Table 4).

**Table 4.** The 10 genes in the MCF7 cell-line predicted by DependANT to have the highest cell-essentiality score together with the experimentally derived Z-score

Gene name	*Z*-score	Likelihood of cell-essentiality
RAD23B	–0.4723	0.9741
RAD23A	0.2654	0.9713
PRPF19	–0.3052	0.9704
SHFM1	–0.3754	0.9681
TP53BP1	0.7196	0.9554
RUVBL2	–0.0575	0.9538
TRIM28	–0.6968	0.9470
XRCC5	–0.2933	0.9467
RAD1	–0.4956	0.9455
XAB2	–0.7499	0.9360

In addition, although only 7 of the 240 genes screened and classified in the MCF7 cell-line had a mean z-score of less than –1 in all 3 repeats, two of these, MEN1 and CHEK1 were predicted as cell-essential with a score of over 0.85 (see Table 5).

**Table 5.** The 10 genes with lowest *Z*-scores in the MCF7 cell line, together with the predicted likelihood of cell-essentiality given by our pan-cancer classifier

Gene name	*Z*-score	Likelihood of cell-essentiality
POLA1	–1.9195	0.6750
MEN1	–1.6886	0.8546
PNKP	–1.5129	0.5851
LIG3	–1.4379	0.3555
CHEK1	–1.2784	0.8503
EME1	–1.2168	0.4798
RBBP8	–1.2160	0.7818
PARP1	–0.9221	0.8987
ERCC2	–0.9021	0.6446
RECQL5	–0.8604	0.5324

### 3.8 Therapeutic opportunities in cancer cell-essential genes

Being able to predict cell-specific dependency genes from genetic analysis of clinical samples allows the direct identification of promising points of therapeutic intervention specific to that sample. Targeting the protein products of tumour-specific dependent genes should kill the cancer cells, whist leaving the normal cells which are not dependent on these genes for viability, relatively unharmed. To explore whether this is a feasible approach we undertook a druggabilty analysis on a set of cell line samples.

Using Cansar’s cancer protein annotation tools (Bulusu *et al*., 2014) we labelled cell-essential genes, based on their respective protein products, as either a druggable target or non-druggable using predictions based on their protein structure. Potential druggable targets were then identified for each of the cell lines in our original training sets.

Next, we identified 35 cell-lines previously unlabelled for gene dependency, 15 for breast, 12 for kidney and 8 for pancreas. Each of these cell-lines were chosen based on the amount of mutation and expression training data available. We used our pan-tissue training set to train our classifiers and produced a full set of dependency predictions for each of these cell-lines (see Supplementary Table S3).

**Fig. 3.**
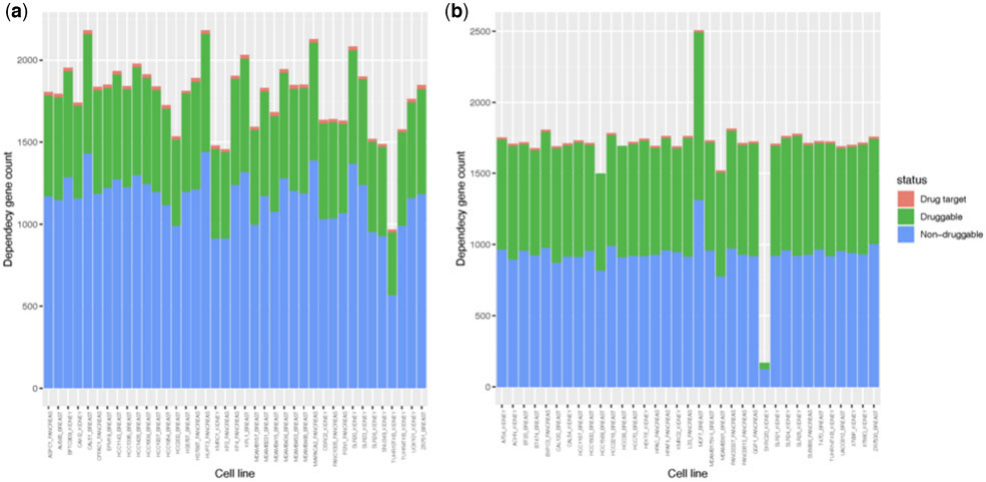
Druggability assessments for (**a**) cell-essential genes in the original training set and (**b**) for the genes predicted to be essential in the unlabelled dataset. Genes who protein products are known drug target are shown in red. Genes whose protein products are predicted to be druggable are shown in green, and those that are non-druggable shown in blue

A druggability analysis of the genes predicted to be cell-essential identified 0.7% whose protein products were known drug targets. The proportion of predicted druggable genes was higher at 45.1% compared to 34.2% in our training set. We found therapeutic opportunities in almost every cell-line in both our training data and prediction set both in the form of genes with known drugs and genes those who exhibit druggable traits (Fig. 3).

## 4 Discussion

PPI maps provide us with a robust model of how the proteome is organised. Here we find that the topological relationships across these maps tends to be different for essential genes and non-essential genes, opening up the opportunity for predicting which genes are essential. However, the real therapeutic benefit would come from using the mutational and expression data associated with a specific cancer cell to predict which genes acquire cell-essentiality as a result of the cancer. The proteins associated with these genes could then be inhibited leading to death of the cancer cells.

A standard PPI map cannot predict whether a gene which is not normally essential will become so under the specific combinations of mutations and changes in gene expression that occur in cancer cells. To address this problem, we first ‘personalized’ the PPI to incorporate information about mutations and gene expression before using resulting topological features to train a random-forest classifier, DependANT. We find that these alterations to the PPI can improve predictions for cell essentiality in human cell-lines with ROC AUC scores of up to 0.84. This is an improvement of 10.8% when compared to the baseline PPI classifier, and an improvement on the accuracy reported by previous studies that use PPI network models to predict essential genes in *S.cerevisiae* (Saha and Heber, 2006) and *E.coli* (da Silva *et al.*, 2008). These improvements are particularly noticeable for genes which are predicted to be essential in only a few cell-lines, a feature which would be useful when looking for therapeutic targets in cancer cells.

Despite the relatively high performance of our classifiers, we are aware that the association between gene expression and protein expression is only partial and so it is likely that further improvements will be possible for this type of model when it is possible to modify the PPI network as a result of protein expression as well as existing ‘omic data. It is possible that improvements to the completeness of our source PPI networks could also lead to significant improvements in this type of study. In particular our source PPI network provides only non-directional, binary information about interactions between proteins rather than the inhibitory or excitatory nature of the interaction. Although we report that our models are relatively robust to incompleteness in the source networks, we expect that as the completeness and sophistication of PPI models improves so will the effectiveness of this type of model. However, it is possible that such improvements may be limited, perhaps because genes with high dependency scores tend to be better studied than genes with lower scores.

The ability to identify therapeutic vulnerabilities for cancer cells using readily available omic data from cancer samples may lead to novel treatment protocols for hard to treat tumours.

## Supplementary Material

vbac084_Supplementary_Data

## References

[vbac084-B2] Aguirre A.J. et al (2016) Genomic copy number dictates a gene-independent cell response to CRISPR/Cas9 targeting. Cancer Discov., 6, 914–929. 27260156 10.1158/2159-8290.CD-16-0154 PMC4972686

[vbac084-B3] Aksoy B.A. et al (2014) Prediction of individualized therapeutic vulnerabilities in cancer from genomic profiles. Bioinformatics, 30, 2051–2059. 24665131 10.1093/bioinformatics/btu164 PMC4080742

[vbac084-B5] Bartha I. et al (2018) Human gene essentiality. Nat. Rev. Genet., 19, 51–62. 29082913 10.1038/nrg.2017.75

[vbac084-B6] Benstead-Hume G. et al (2017) ‘Big data’ approaches for novel anti-cancer drug discovery. Expert Opin. Drug Discov., 12, 599–609. 28462602 10.1080/17460441.2017.1319356

[vbac084-B7] Birmingham A. et al (2006) 3’ UTR seed matches, but not overall identity, are associated with RNAi off-targets. Nat. Methods, 3, 199–204. 16489337 10.1038/nmeth854

[vbac084-B8] Buehler E. et al (2012) C911: a bench-level control for sequence specific siRNA off-target effects. PLoS One, 7, e51942. 23251657 10.1371/journal.pone.0051942 PMC3522603

[vbac084-B9] Burley S.K. et al (2022) RCSB protein data bank: celebrating 50 years of the PDB with new tools for understanding and visualizing biological macromolecules in 3D. Protein Sci., 31, 187–208. 34676613 10.1002/pro.4213 PMC8740825

[vbac084-B49] Bulusu K.C. et al (2014) canSAR: updated cancer research and drug discovery knowledgebase. Nucleic Acids Res., 42, D1040–D1047. 24304894 10.1093/nar/gkt1182 PMC3964944

[vbac084-B10] Charlton,P. and Spicer,J. (2016) Targeted therapy in cancer. Medicine, 44, 34–38.

[vbac084-B11] Chen Y. , Xu D. (2005) Understanding protein dispensability through machine-learning analysis of high-throughput data. Bioinformatics, 21, 575–581. 15479713 10.1093/bioinformatics/bti058

[vbac084-B12] Cheng J. et al (2014) Training set selection for the prediction of essential genes. PLoS One, 9, e86805. 24466248 10.1371/journal.pone.0086805 PMC3899339

[vbac084-B13] Cheung H.W. et al (2011) Systematic investigation of genetic vulnerabilities across cancer cell lines reveals lineage-specific dependencies in ovarian cancer. Proc. Natl. Acad. Sci. USA, 108, 12372–12377. 21746896 10.1073/pnas.1109363108 PMC3145679

[vbac084-B14] Coker E.A. et al (2019) CanSAR: update to the cancer translational research and drug discovery knowledgebase. Nucleic Acids Res., 47, D917–D922. 30496479 10.1093/nar/gky1129 PMC6323893

[vbac084-B15] Csárdi G. , Nepusz T. (2006) The igraph software package for complex network research. Int. J. Complex Syst., 1695, 1–9.

[vbac084-B16] Dai W. et al (2020) Network embedding the protein–protein interaction network for human essential genes identification. Genes (Basel), 11, 153. 32023848 10.3390/genes11020153 PMC7074227

[vbac084-B17] Eisenberg E. , Levanon E.Y. (2013) Human housekeeping genes, revisited. Trends Genet., 29, 569–574. 23810203 10.1016/j.tig.2013.05.010

[vbac084-B18] Hubbard T. et al (2002) The ensembl genome database project. Nucleic Acids Res., 30, 38–41. 11752248 10.1093/nar/30.1.38 PMC99161

[vbac084-B19] Huttlin E.L. et al (2017) Architecture of the human interactome defines protein communities and disease networks. Nature, 545, 505–509. 28514442 10.1038/nature22366 PMC5531611

[vbac084-B20] Hwang Y.C. et al (2009) Predicting essential genes based on network and sequence analysis. Mol. Biosyst., 5, 1672–1678. 19452048 10.1039/B900611G

[vbac084-B21] Jackson A.L. , Linsley P.S. (2004) Noise amidst the silence: off-target effects of siRNAs? Trends Genet., 20, 521–524. 15475108 10.1016/j.tig.2004.08.006

[vbac084-B22] Jacunski A. et al (2015) Connectivity Homology Enables Inter-Species Network Models of Synthetic Lethality. *PLoS Comput Biol.*, **11**, e1004506 10.1371/journal.pcbi.1004506 PMC4599967 26451775

[vbac084-B23] Lu Y. et al (2014) Predicting essential genes for identifying potential drug targets in *Aspergillus fumigatus*. Comput. Biol. Chem., 50, 29–40. 24569026 10.1016/j.compbiolchem.2014.01.011

[vbac084-B24] Luo B. et al (2008) Highly parallel identification of essential genes in cancer cells. Proc. Natl. Acad. Sci. USA, 105, 20380–20385. 19091943 10.1073/pnas.0810485105 PMC2629277

[vbac084-B25] Marcotte R. et al (2012) Essential gene profiles in breast, pancreatic, and ovarian cancer cells. Cancer Discov., 2, 172–189. 22585861 10.1158/2159-8290.CD-11-0224 PMC5057396

[vbac084-B26] Marcotte R. et al (2016) Functional genomic landscape of human breast resource functional genomic landscape of human breast cancer drivers, vulnerabilities, and resistance. Cell, 164, 293–309. 26771497 10.1016/j.cell.2015.11.062 PMC4724865

[vbac084-B28] Meyers,R.M. et al (2017) Computational correction of copy number effect improves specificity of CRISPR-Cas9 essentiality screens in cancer cells. Nat. Genet., 49, 1779–1784. 29083409 10.1038/ng.3984 PMC5709193

[vbac084-B29] Mosca R. et al (2013) Towards a detailed atlas of protein–protein interactions. Curr. Opin. Struct. Biol., 23, 929–940. 23896349 10.1016/j.sbi.2013.07.005

[vbac084-B30] Munoz D.M. et al (2016) CRISPR screens provide a comprehensive assessment of cancer vulnerabilities but generate false-positive hits for highly amplified genomic regions. Cancer Discov., 6, 900–913. 27260157 10.1158/2159-8290.CD-16-0178

[vbac084-B31] Ngo V.N. et al (2006) A loss-of-function RNA interference screen for molecular targets in cancer. Nature, 441, 106–110. 16572121 10.1038/nature04687

[vbac084-B32] Plaimas K. et al (2010) Identifying essential genes in bacterial metabolic networks with machine learning methods. BMC Syst. Biol., 4, 56. 20438628 10.1186/1752-0509-4-56 PMC2874528

[vbac084-B33] Rolland T. et al (2014) A proteome-scale map of the human interactome network. Cell, 159, 1212–1226. 25416956 10.1016/j.cell.2014.10.050 PMC4266588

[vbac084-B34] Roumeliotis T.I. et al (2017) Genomic determinants of protein abundance variation in colorectal cancer cells. Cell Rep., 20, 2201–2214. 28854368 10.1016/j.celrep.2017.08.010 PMC5583477

[vbac084-B35] Saha S. , Heber S. (2006) In silico prediction of yeast deletion phenotypes. Genet. Mol. Res., 5, 224–232. 16755513

[vbac084-B36] Schapke J. et al (2021) EPGAT: gene essentiality prediction with graph attention networks. *IEEE/ACM Trans. Comput. Biol. Bioinform*., **19**, 1615–1626. 10.1109/TCBB.2021.3054738 33497339

[vbac084-B37] Shihab H.A. et al (2013) Predicting the functional, molecular, and phenotypic consequences of amino acid substitutions using hidden Markov models. Hum. Mutat., 34, 57–65. 23033316 10.1002/humu.22225 PMC3558800

[vbac084-B38] da Silva J.P.M. et al (2008) In silico network topology-based prediction of gene essentiality. Phys. A Stat. Mech. Appl., 387, 1049–1055.

[vbac084-B39] Tsherniak A. et al (2017) Defining a cancer dependency map. Cell, 170, 564–576.e16. 28753430 10.1016/j.cell.2017.06.010 PMC5667678

[vbac084-B27] Von Mering,C. et al (2005) STRING: known and predicted protein-protein associations, integrated and transferred across organisms. Nucleic Acids Res., 33, D433–D437. 15608232 10.1093/nar/gki005 PMC539959

[vbac084-B40] Weinstein I.B. (2002) Cancer: addiction to oncogenes – the achilles heal of cancer. Science, 297, 63–64. 12098689 10.1126/science.1073096

[vbac084-B41] Workman,P. et al (2013) Genome-based cancer therapeutics: targets, kinase drug resistance and future strategies for precision oncology. Curr. Opin. Pharmacol., 13, 486–496. 23810823 10.1016/j.coph.2013.06.004

[vbac084-B43] Zhang X. et al (2016) Predicting essential genes and proteins based on machine learning and network topological features: A comprehensive review. Front. Physiol., 7, 1–11. 27014079 10.3389/fphys.2016.00075 PMC4781880

[vbac084-B42] Zhang X. et al (2020) DeepHE: accurately predicting human essential genes based on deep learning. PLoS Comput. Biol., 16, e1008229. 32936825 10.1371/journal.pcbi.1008229 PMC7521708

