# Supplementary material for: Biological network topology features predict gene dependencies in cancer cell-lines
Source: Bioinform Adv. 2022 Nov 10;2(1):vbac084. doi: 10.1093/bioadv/vbac084 (PMC9681200; doi:10.1093/bioadv/vbac084)
Supplement: vbac084_Supplementary_Data [file vbac084_supplementary_data.zip › Supplementary File Final.docx]

# Biological network topology features predict gene dependencies in cancer cell-lines

# Supplementary Data

Graeme Benstead-Hume^1,2,*^, Sarah K. Wooller^1,*^, Joanna Renaut^1^, Samantha Dias^3^, Lisa Woodbine^3^, Antony M. Carr^3^, Frances M. G. Pearl^1,$^

^1^Bioinformatics Lab, School of Life Sciences, University of Sussex, Brighton. BN1 9QJ, United Kingdom, ^2^ Division of Cancer Biology, The Institute of Cancer Research, London SW3 6JB, United Kingdom, ^3^Genome Damage and Stability Centre, University of Sussex, Brighton BN1 9RQ, United Kingdom

* Contributed equally to the work, ^$^ To whom correspondence should be addressed

**Supplementary Figure S1**. Feature Importance

*Note:* To quantify which features provide the most predictive power to our models we calculated a normalised importance score for each feature for each cell line and took the distribution of these scores across all cell lines. Feature importance was calculated by measuring the mean decrease in accuracy without each feature across all tree permutations in a random forest.

We found that a number of features that measure connectivity of a gene perform better than degree centrality although degree centrality does provide a moderate amount of predictive power. Page rank and eigen centrality scored well in all cell line models followed by hub score and constraint. Eccentricity, the distance a given node is away from the furthest node from itself in the network, a measure of how close that node is to the centre of the network, performs badly across all models.

These importance scores reflected the class feature distributions fairly well, i.e. features whose values varied more between essential and non-essential genes provided more predictive power.

Pagerank and constraint showed a noticeable differentiation between classes while the differentiation between classes for eigen centrality and hubscore features were not as prominent.

**Supplementary Figure S2**. Scatter plot for the consensus Z-score from the experimental validation versus the DependANT prediction score.

**Supplementary Figure S3**. Scatter plot of the MCF7 gene effect score from DepMap 22Q1 versus the DependANT prediction score. DepMap scores for MCF7 have only recently become available.

**Supplementary Figure S4**. Scatter plot of the MCF7 gene effect score from DepMap 22Q1 versus the DependANT prediction score. Pan-cell line essential genes are shown in orange.

**Supplementary Tables S1**. Cell line details used to generate features for dependANT.

| **Cell line name (CCLE)** | **DepMap ID.** | **No. of mutations** | **No of dependent genes** |
| --- | --- | --- | --- |
| ASPC1_PANCREAS | ACH-000222 | 304 | 1566 |
| AU565_BREAST | ACH-000248 | 799 | 1601 |
| BFTC909_KIDNEY | ACH-000792 | 442 | 1689 |
| CAKI2_KIDNEY | ACH-000234 | 267 | 1453 |
| CAL51_BREAST | ACH-000856 | 1134 | 1913 |
| CFPAC1_PANCREAS | ACH-000138 | 203 | 1649 |
| EFM19_BREAST | ACH-000330 | 337 | 1561 |
| HCC1143_BREAST | ACH-000374 | 287 | 1581 |
| HCC1395_BREAST | ACH-000699 | 578 | 1643 |
| HCC1428_BREAST | ACH-000352 | 465 | 1728 |
| HCC1806_BREAST | ACH-000624 | 393 | 1680 |
| HCC1937_BREAST | ACH-000223 | 366 | 1599 |
| HCC1954_BREAST | ACH-000859 | 502 | 1499 |
| HCC202_BREAST | ACH-000725 | 633 | 1349 |
| HS578T_BREAST | ACH-000148 | 244 | 1571 |
| HS766T_PANCREAS | ACH-000178 | 273 | 1653 |
| HUPT3_PANCREAS | ACH-000118 | 300 | 1941 |
| KMRC1_KIDNEY | ACH-000684 | 381 | 1251 |
| KP2_PANCREAS | ACH-000281 | 350 | 1251 |
| KP4_PANCREAS | ACH-000265 | 360 | 1637 |
| KPL1_BREAST | ACH-000028 | 490 | 1734 |
| MDAMB157_BREAST | ACH-000621 | 660 | 1411 |
| MDAMB231_BREAST | ACH-000768 | 580 | 1609 |
| MDAMB415_BREAST | ACH-000876 | 1013 | 1555 |
| MDAMB436_BREAST | ACH-000573 | 465 | 1709 |
| MDAMB453_BREAST | ACH-000910 | 1201 | 1595 |
| MDAMB468_BREAST | ACH-000849 | 575 | 1675 |
| MIAPACA2_PANCREAS | ACH-000601 | 456 | 1880 |
| OSRC2_KIDNEY | ACH-000159 | 368 | 1434 |
| PANC1005_PANCREAS | ACH-000060 | 231 | 1464 |
| PSN1_PANCREAS | ACH-000320 | 313 | 1461 |
| SLR20_KIDNEY | ACH-000127 | 436 | 1829 |
| SLR23_KIDNEY | ACH-000246 | 326 | 1671 |
| SLR26_KIDNEY | ACH-000600 | 229 | 1287 |
| SNU349_KIDNEY | ACH-000907 | 1305 | 1275 |
| TUHR10TKB_KIDNEY | ACH-000459 | 332 | 836 |
| TUHR4TKB_KIDNEY | ACH-000495 | 313 | 1377 |
| UOK101_KIDNEY | ACH-000262 | 260 | 1572 |
| ZR751_BREAST | ACH-000097 | 360 | 1647 |

*Notes:* Data on each of cell lines used to generate the PPI. The standardised PPI contained 7262 proteins/nodes. Mutated proteins were labelled as neutral, gain or loss of function. Each edge was labelled with a value derived from the expression data for the connecting genes (see methods).

**Supplementary Table S2:** Survival screens Z-scores for 240 genes involved in the DNA damage response (DDR), in the MCF7 breast cell-line.

| **Gene** | **Repeat 1** | **Repeat 2** | **Repeat 3** | **Mean** |
| --- | --- | --- | --- | --- |
| ABL1 | -0.70245 | -0.68634 | -1.17973 | -0.85618 |
| ADPRTL3 | -0.81644 | -0.02941 | -1.02285 | -0.62290 |
| ALKBH | -0.18206 | -0.51542 | 0.09230 | -0.20173 |
| ALKBH2 | 0.30273 | 0.97447 | 0.47312 | 0.58344 |
| APEX1 | -0.42380 | 0.33872 | 0.04151 | -0.01453 |
| APEX2 | 0.24070 | -0.10475 | 0.64514 | 0.26036 |
| APTX | 0.59860 | 2.65331 | 0.46755 | 1.23982 |
| ASF1A | 1.93896 | 0.77956 | 0.88944 | 1.20265 |
| ATF2 | -0.23374 | 1.14933 | -2.09682 | -0.39374 |
| ATM | -0.41673 | -0.64985 | -0.10975 | -0.39211 |
| ATR | 0.33467 | 2.04393 | 1.12419 | 1.16759 |
| ATRIP | 0.19200 | -1.08435 | 0.77674 | -0.03854 |
| ATRX | -0.00391 | 0.13782 | -0.31810 | -0.06140 |
| BAZ1B | -1.39813 | -1.52538 | -1.64008 | -1.52120 |
| BLM | 0.50612 | -0.55605 | 1.89677 | 0.61562 |
| BRCA1 | 0.07783 | 0.51719 | 0.00572 | 0.20025 |
| BRCA2 | 0.43171 | 1.22399 | -0.61356 | 0.34738 |
| BRE | 0.53458 | 0.18650 | 0.26333 | 0.32814 |
| BRIP1 | -0.15655 | 1.17325 | 0.72623 | 0.58098 |
| BTG2 | 0.27707 | -0.47925 | 1.17434 | 0.32405 |
| C11ORF13 | -0.44737 | -0.11267 | -0.84550 | -0.46852 |
| C2ORF13 | 1.58435 | -0.49068 | 0.86842 | 0.65403 |
| C7ORF11 | -0.00302 | -0.53142 | -0.29747 | -0.27730 |
| CCNH | -1.42392 | -0.79674 | 0.16075 | -0.68664 |
| CDK7 | 0.77675 | 0.03116 | 1.06125 | 0.62305 |
| CDKN2D | -0.79823 | -0.90264 | -0.26315 | -0.65467 |
| CETN2 | 0.44580 | -0.77357 | 0.76857 | 0.14694 |
| CHAF1A | 1.91717 | 0.26242 | -0.52276 | 0.55227 |
| CHEK1 | -1.28052 | -1.66965 | -0.88509 | -1.27842 |
| CHEK2 | -0.91725 | -1.00571 | -0.15561 | -0.69286 |
| CIB1 | 0.17741 | -0.43264 | 0.51112 | 0.08530 |
| CKN1 | -0.20532 | -1.45673 | -1.10119 | -0.92108 |
| CLK2 | 0.41631 | 0.00292 | -0.80573 | -0.12883 |
| CNOT7 | 0.14079 | -0.87970 | 0.50686 | -0.07735 |
| CSNK1D | 0.35551 | 0.72703 | -0.26548 | 0.27235 |
| CSNK1E | 0.71573 | 0.72967 | 0.62657 | 0.69066 |
| CSPG6 | -0.41512 | 0.07893 | -0.06021 | -0.13213 |
| CXORF53 | 0.61920 | 0.12328 | 0.61434 | 0.45227 |
| DCLRE1A | 0.15106 | 1.00217 | 0.41474 | 0.52266 |
| DCLRE1B | 0.04127 | -1.23864 | -0.17108 | -0.45615 |
| DCLRE1C | 0.30440 | 0.25446 | 0.53744 | 0.36543 |
| DDB1 | 0.39147 | -0.20161 | 1.01367 | 0.40118 |
| DDB2 | -0.21664 | -0.69858 | 0.66171 | -0.08450 |
| DDX11 | 0.39030 | 1.40527 | 1.01171 | 0.93576 |
| DEPC-1 | 0.08208 | -0.07070 | 1.23452 | 0.41530 |
| DLG7 | -1.13636 | -1.10790 | -0.17331 | -0.80586 |
| DMC1 | -0.40678 | 0.26665 | -0.29920 | -0.14644 |
| DNA2L | 0.30121 | 1.32476 | 1.16898 | 0.93165 |
| DNMT1 | 0.10163 | 0.84124 | 0.73884 | 0.56057 |
| DUT | -0.15977 | -0.38888 | -0.67229 | -0.40698 |
| EME1 | -1.11833 | -1.28464 | -1.24742 | -1.21680 |
| EME2 | 0.36823 | -0.32080 | 0.62087 | 0.22277 |
| ERCC1 | 0.99519 | 1.05165 | 1.01822 | 1.02169 |
| ERCC2 | -0.64859 | -1.46640 | -0.59127 | -0.90209 |
| ERCC3 | -0.54222 | -0.48355 | 0.69081 | -0.11165 |
| ERCC4 | 1.03498 | -0.28201 | 0.78953 | 0.51416 |
| ERCC5 | 0.73908 | 1.22854 | 0.04105 | 0.66956 |
| ERCC6 | 1.95777 | 0.12328 | 1.40672 | 1.16259 |
| EXO1 | -0.21772 | -0.06374 | -1.50804 | -0.59650 |
| EYA1 | 0.04286 | 0.28751 | -0.77365 | -0.14776 |
| EYA3 | 0.96560 | -0.97567 | 0.53349 | 0.17447 |
| FANCA | -0.27734 | -0.34164 | -0.52189 | -0.38029 |
| FANCB | 0.18720 | -0.02943 | 0.55368 | 0.23715 |
| FANCC | 0.37703 | 1.41544 | 0.27448 | 0.68899 |
| FANCD2 | -1.25918 | 0.32482 | -0.84405 | -0.59281 |
| FANCE | 0.01041 | -1.38544 | 0.12003 | -0.41833 |
| FANCF | -0.24133 | -0.67241 | 0.19327 | -0.24016 |
| FANCG | -0.49473 | 1.31195 | 0.62444 | 0.48055 |
| FANCL | -0.67844 | 0.44803 | -1.05146 | -0.42729 |
| FEN1 | -0.07198 | 1.13464 | 0.05532 | 0.37266 |
| FLJ10719 | 1.41786 | -0.44574 | 0.58738 | 0.51983 |
| FLJ12610 | -0.11479 | -0.21166 | 0.63995 | 0.10450 |
| FLJ13614 | 0.18445 | 0.35403 | 0.22021 | 0.25290 |
| FLJ21816 | 0.03858 | -1.03304 | 0.47139 | -0.17436 |
| FLJ22833 | 2.82488 | 1.38412 | 2.73015 | 2.31305 |
| FLJ40869 | 1.36544 | 0.62388 | 0.73180 | 0.90704 |
| FRAP1 | 0.39223 | -0.09626 | -0.40453 | -0.03619 |
| G22P1 | -0.39531 | -1.01192 | -0.51976 | -0.64233 |
| GADD45A | 0.48820 | 0.17888 | -0.02640 | 0.21356 |
| GADD45G | 1.37903 | -0.13483 | -0.25743 | 0.32892 |
| GCN5L2 | 0.50005 | 1.86437 | 0.33675 | 0.90039 |
| GIYD1 | -1.57001 | -2.18032 | -1.42822 | -1.72618 |
| GTF2H1 | 1.11671 | 1.30294 | 0.56659 | 0.99541 |
| GTF2H2 | 0.88477 | 1.18682 | 0.47258 | 0.84806 |
| GTF2H3 | -0.66088 | -0.98461 | -0.78014 | -0.80854 |
| GTF2H4 | -0.04060 | 1.66104 | 0.96951 | 0.86332 |
| GTF2H5 | -0.82692 | 0.48990 | -0.22931 | -0.18877 |
| H2AFX | 2.79494 | 2.38560 | 1.72631 | 2.30229 |
| HEL308 | 0.08214 | 0.82350 | -0.04044 | 0.28840 |
| HMGB1 | 0.08662 | -0.05852 | -0.58660 | -0.18617 |
| HMGB2 | 1.02240 | 1.31463 | 0.21565 | 0.85089 |
| HRMT1L6 | 1.99290 | -0.14035 | 1.81640 | 1.22298 |
| HSU24186 | 0.36347 | 0.09634 | -0.84856 | -0.12958 |
| HTATIP | 0.40520 | 0.84124 | 1.04911 | 0.76519 |
| HUS1 | -0.21200 | 1.41801 | 0.22931 | 0.47844 |
| IGHMBP2 | -0.27284 | -0.36381 | -0.19405 | -0.27690 |
| IHPK3 | -1.21263 | -1.04813 | -0.92204 | -1.06094 |
| KIAA0625 | -0.41933 | -1.17888 | -0.40812 | -0.66878 |
| KIAA1018 | -0.83123 | -1.70197 | -0.90176 | -1.14499 |
| KIAA1596 | -1.31029 | 0.63002 | 0.56492 | -0.03845 |
| KUB3 | -0.51389 | -1.34256 | -1.34315 | -1.06653 |
| LIG1 | 2.05116 | 0.82902 | 1.59215 | 1.49078 |
| LIG3 | -0.76366 | -1.70311 | -1.84692 | -1.43790 |
| LIG4 | 0.24912 | 1.05996 | 0.07514 | 0.46141 |
| MAD2L2 | -0.43280 | -0.42420 | -0.87526 | -0.57742 |
| MBD4 | 0.23275 | -0.23249 | -0.49888 | -0.16621 |
| MDC1 | -0.25619 | 0.52992 | 0.97264 | 0.41546 |
| MEN1 | -1.17657 | -1.50423 | -2.38497 | -1.68859 |
| MGC2731 | 1.31549 | -0.62804 | -0.46940 | 0.07268 |
| MGC32020 | 0.32608 | -0.53548 | 1.63919 | 0.47660 |
| MGC4189 | 0.19470 | -0.09555 | 0.56577 | 0.22164 |
| MGMT | 1.02585 | -0.72126 | -0.10152 | 0.06769 |
| MIZF | -1.62034 | -0.92140 | -0.35315 | -0.96497 |
| MJD | -0.04087 | 0.19715 | 0.91327 | 0.35652 |
| MLH1 | 1.45431 | -0.92158 | -0.26259 | 0.09005 |
| MLH3 | 1.07741 | 2.03176 | 1.61051 | 1.57323 |
| MMS19L | 0.39951 | -0.51855 | -0.17624 | -0.09842 |
| MNAT1 | 0.66002 | -0.36193 | -0.33683 | -0.01291 |
| MPG | -0.72463 | 0.15995 | 0.35811 | -0.06886 |
| MRE11A | -1.21080 | -0.96397 | 0.44052 | -0.57808 |
| MSH2 | 0.39810 | 1.18171 | 2.02904 | 1.20295 |
| MSH3 | -0.48130 | 1.45713 | 0.83505 | 0.60363 |
| MSH4 | -0.14970 | 1.35534 | 0.71669 | 0.64078 |
| MSH5 | -0.66960 | 0.27127 | 0.26905 | -0.04309 |
| MSH6 | -0.08075 | -0.49164 | -0.43280 | -0.33506 |
| MUS81 | 0.10984 | 1.86437 | 0.69640 | 0.89020 |
| MUTYH | -0.12433 | -1.01332 | -0.40127 | -0.51297 |
| NBS1 | -1.43169 | -0.55210 | -0.35841 | -0.78073 |
| NEIL1 | 0.47935 | 0.33703 | 1.44444 | 0.75361 |
| NEIL2 | -0.32001 | 1.23950 | 0.40230 | 0.44060 |
| NEIL3 | -0.05293 | -0.70769 | -0.10638 | -0.28900 |
| NPM1 | 1.10725 | 0.15147 | 0.48970 | 0.58281 |
| NTHL1 | 1.58097 | 0.49370 | -0.75204 | 0.44087 |
| NUDT1 | -0.19498 | -0.79031 | -0.35642 | -0.44724 |
| OGG1 | 0.75985 | -0.11026 | -0.73516 | -0.02852 |
| PARG | -1.23079 | -0.32616 | -0.60407 | -0.72034 |
| PARP1 | -0.52939 | -2.79967 | 0.56267 | -0.92213 |
| PARP2 | 0.44408 | 0.46899 | 0.36247 | 0.42518 |
| PCNA | -0.30034 | -0.09669 | -0.03717 | -0.14473 |
| PER1 | -0.03244 | 1.29852 | 0.70609 | 0.65739 |
| PMS1 | -0.18319 | 0.32149 | -0.20107 | -0.02092 |
| PMS2 | -0.32181 | 1.18077 | 0.32317 | 0.39405 |
| PMS2L5 | -0.41130 | -0.71439 | -0.73748 | -0.62106 |
| PNKP | -2.35301 | -1.01001 | -1.17571 | -1.51291 |
| POL2G | 0.30143 | 0.82902 | 0.36250 | 0.49765 |
| POLA 1 | -1.84890 | -1.90455 | -2.00512 | -1.91952 |
| POLB | -0.19940 | -0.28214 | 0.32022 | -0.05377 |
| POLD1 | -0.35532 | -0.99750 | -0.39630 | -0.58304 |
| POLE | -1.08723 | 0.52942 | 0.13957 | -0.13941 |
| POLE2 | -0.40954 | 1.45633 | 0.31035 | 0.45238 |
| POLG | 0.57217 | 0.37089 | 0.49164 | 0.47823 |
| POLH | 0.76086 | -0.39889 | -0.71222 | -0.11675 |
| POLI | 0.43362 | 0.92222 | 0.16567 | 0.50717 |
| POLK | -0.49260 | -0.44376 | -0.46425 | -0.46687 |
| POLL | 0.62445 | -0.58475 | 0.38272 | 0.14081 |
| POLM | -0.51544 | -0.10287 | -0.01717 | -0.21183 |
| POLN | -0.50544 | 0.29299 | 0.10052 | -0.03731 |
| POLQ | -0.46212 | -1.18553 | -1.58271 | -1.07679 |
| POLS | -0.04025 | -0.39732 | -0.78852 | -0.40870 |
| PRKCG | 0.12432 | 1.18682 | -0.95783 | 0.11777 |
| PRKDC | -0.50499 | 0.65643 | 0.52409 | 0.22518 |
| PRPF19 | -0.25014 | 0.01275 | -0.67820 | -0.30520 |
| RAD1 | -1.50065 | -0.12103 | 0.13503 | -0.49555 |
| RAD17 | -0.70120 | -0.12313 | -0.15605 | -0.32679 |
| RAD18 | 0.09287 | 1.05097 | -0.31332 | 0.27684 |
| RAD21 | 1.22333 | 0.12285 | 0.63506 | 0.66041 |
| RAD23A | 0.83630 | -0.42037 | 0.38037 | 0.26543 |
| RAD23B | -0.13989 | -0.86336 | -0.41364 | -0.47230 |
| RAD50 | -0.09130 | 2.25341 | -0.63352 | 0.50953 |
| RAD51 | -0.08929 | 0.62329 | 2.18312 | 0.90571 |
| RAD51C | 1.38139 | -0.24049 | 1.11552 | 0.75214 |
| RAD51L1 | 0.17307 | -0.32175 | -0.28234 | -0.14367 |
| RAD51L3 | 0.68646 | 1.85732 | -0.08432 | 0.81982 |
| RAD52 | -0.50446 | 0.28711 | -0.74653 | -0.32129 |
| RAD52B | 0.89227 | -0.94169 | 0.00586 | -0.01452 |
| RAD54B | 0.81271 | -0.39732 | 0.30210 | 0.23916 |
| RAD54L | 0.09356 | -0.33551 | -0.03388 | -0.09195 |
| RAD9A | 0.71677 | 0.81671 | 1.03712 | 0.85687 |
| RAP80 | 0.23419 | 2.65331 | 0.27456 | 1.05402 |
| RBBP8 | -1.81933 | -1.13707 | -0.69169 | -1.21603 |
| RECQL | 0.73291 | 1.08348 | 0.91628 | 0.91089 |
| RECQL4 | -0.47992 | -0.86803 | -0.87298 | -0.74031 |
| RECQL5 | -1.75870 | -0.07648 | -0.74608 | -0.86042 |
| RENT1 | 0.63557 | -0.55142 | -0.03979 | 0.01479 |
| REV1L | -0.67565 | 0.76492 | 1.03498 | 0.37475 |
| REV3L | -0.58742 | -0.16095 | -0.34408 | -0.36415 |
| RNF168 | -0.15150 | -1.04902 | -0.22126 | -0.47393 |
| RNF8 | 0.63712 | -0.45115 | 0.74709 | 0.31102 |
| RPA1 | -1.12590 | -0.49213 | 0.28196 | -0.44535 |
| RPA2 | 1.51438 | 0.28829 | 1.16700 | 0.98989 |
| RPA3 | 1.51037 | 1.00217 | 0.51776 | 1.01010 |
| RPS27L | -0.96228 | 1.02756 | 1.18790 | 0.41773 |
| RRM2 | -0.35149 | -0.53645 | 0.79457 | -0.03113 |
| RRM2B | 1.89507 | -0.02917 | 0.77029 | 0.87873 |
| RTEL1 | -0.21119 | 1.22854 | -0.76713 | 0.08340 |
| RUVBL2 | 0.06540 | 0.42390 | -0.66168 | -0.05746 |
| Scrambled 1D1 | -0.22632 | 0.65430 | 0.38681 | 0.27159 |
| Scrambled 1D12 | -0.03278 | -0.89217 | 0.02182 | -0.30104 |
| Scrambled 2D1 | -0.02307 | -0.39263 | -0.17529 | -0.19700 |
| Scrambled 2D12 | -0.23603 | 0.15476 | 0.58393 | 0.16755 |
| Scrambled 3D1 | 0.09784 | -0.15674 | 0.52759 | 0.15623 |
| Scrambled 3D12 | -0.35695 | -0.08113 | -0.11896 | -0.18568 |
| SETMAR | -0.72024 | 0.81160 | -0.39482 | -0.10115 |
| SHFM1 | -0.05497 | -0.73543 | -0.33588 | -0.37543 |
| SIRT1 | -0.06705 | -0.68391 | 0.59735 | -0.05121 |
| SMC1L1 | 1.45509 | -0.36364 | 0.78681 | 0.62609 |
| SMC6L1 | 0.48951 | 0.24637 | 0.05344 | 0.26311 |
| SMUG1 | 0.51729 | -0.01514 | 0.14094 | 0.21436 |
| SOD1 | -0.14594 | 0.93348 | 0.25754 | 0.34836 |
| SPO11 | 1.48844 | 0.30459 | -0.04976 | 0.58109 |
| TADA3L | -0.77860 | -1.30025 | -5.34244 | -2.47376 |
| TCEA1 | 0.43055 | 1.30294 | 0.34301 | 0.69217 |
| TDG | -0.94600 | 0.77107 | -0.06074 | -0.07856 |
| TDP1 | 0.68911 | 0.76815 | 0.10061 | 0.51929 |
| TNP1 | 0.09759 | -0.45376 | 0.43718 | 0.02700 |
| TOP2A | 0.21794 | 0.85694 | 0.67915 | 0.58468 |
| TOPBP1 | -1.31299 | 0.49537 | -0.40795 | -0.40852 |
| TP53 | 1.37563 | -0.05208 | -0.90718 | 0.13879 |
| TP53BP1 | 1.62291 | -0.07348 | 0.60927 | 0.71957 |
| TP73 | -0.52579 | -1.77394 | -0.26267 | -0.85414 |
| TREX1 | -0.49637 | 0.24402 | -0.85634 | -0.36956 |
| TREX2 | -0.21161 | 0.30459 | 0.15474 | 0.08258 |
| TRIM28 | -0.15975 | -1.23543 | -0.69530 | -0.69682 |
| TRIP13 | 0.13387 | 0.36343 | -0.92113 | -0.14128 |
| TTRAP | -1.62391 | -0.08876 | -1.29245 | -1.00170 |
| TYMS | -0.02047 | -0.07334 | -1.09610 | -0.39664 |
| UBE2A | 0.59513 | -0.54520 | -0.73293 | -0.22767 |
| UBE2B | 0.02142 | 0.81091 | -0.21817 | 0.20472 |
| UBE2N | 4.59005 | 1.34179 | 4.42493 | 3.45226 |
| UBE2V1 | 0.35857 | 0.34091 | 1.70635 | 0.80194 |
| UBE2V2 | 0.89150 | -0.59485 | -0.90990 | -0.20441 |
| UNG | 0.19245 | 0.03074 | 1.54442 | 0.58920 |
| UNG2 | -0.50719 | 0.42390 | -0.60750 | -0.23026 |
| USP1 | 0.29243 | 1.08348 | -0.06539 | 0.43684 |
| UVRAG | 0.11816 | 0.26242 | -1.23515 | -0.28486 |
| VCP | -0.04841 | 0.21666 | -0.00977 | 0.05283 |
| WRN | 0.37470 | 2.31616 | -0.18383 | 0.83568 |
| XAB2 | -1.60645 | -0.48478 | -0.15834 | -0.74986 |
| XPA | 0.58060 | -0.96384 | 0.12097 | -0.08742 |
| XPC | -0.36284 | -0.13916 | 0.24404 | -0.08599 |
| XRCC1 | 0.67291 | 1.13464 | 0.68045 | 0.82933 |
| XRCC2 | 0.01507 | -0.30349 | 0.26060 | -0.00928 |
| XRCC3 | 0.66837 | -0.58883 | 0.48360 | 0.18771 |
| XRCC4 | 0.64032 | 0.82885 | -0.87934 | 0.19661 |
| XRCC5 | -1.19055 | -0.36373 | 0.67440 | -0.29329 |
| YBX1 | 1.13010 | 1.41544 | 0.52623 | 1.02392 |

*Note:* It is common for these screens to be quite variable between repeats (see Results).

Correlations coefficients; Repeats 1 and 2, *r* = 0.3462, *p-value* = 4.0 x 10^-8^. Repeat 1 and 3, *r* = 0.3483, *p-value* = 0.0. Repeats 2 and 3, *r* = 0.2987, *p-value* = 2.65 x 10^-6^.
